# A Quasi-Lumped Element Tunable Bandpass Filter Based on GaAs Technology

**DOI:** 10.3390/mi17030292

**Published:** 2026-02-27

**Authors:** Xulei Cheng, Bin You

**Affiliations:** The Innovation Center for Electronic Design Automation Technology, School of Electronics and Information (School of IC Science and Engineering), Hangzhou Dianzi University, Hangzhou 310018, China; chengxl@hdu.edu.cn

**Keywords:** GaAs technology, tunable filter, quasi-lumped element

## Abstract

This paper presents a miniaturized tunable bandpass filter chip fabricated using a gallium arsenide (GaAs) technology. In the layout design, a quasi-lumped element is utilized to replace conventional spiral inductors, complemented by on-chip PN-junction varactor diodes and Metal-Insulator-Metal (MIM) capacitors. The integration of a source-load coupling structure and grounded series LC resonators introduces three transmission zeros (*TZs*), enhancing the frequency selectivity. By independently tuning the coupling capacitance and the grounded series LC resonant structures, the operating frequency of the filter achieves continuous tunability. An equivalent circuit model is established to analyze the filter’s performance. For experimental verification, the proposed filter was fabricated and measured, occupying a compact die area of 1.35 × 1.365 mm^2^. The measured results demonstrate a center frequency tuning range from 5.4 to 6.2 GHz, showing good agreement with simulation and thus validating the proposed miniaturized continuously tunable filter.

## 1. Introduction

With the continuous miniaturization of wireless terminal devices and the increasing number of communication frequency bands, there is a growing demand for compact and multiband filters in modern wireless systems. Tunable filters, as an effective solution for realizing multiband operation, have attracted considerable attention. At present, research on tunable filters is mainly focused on varactor-diode-tuned filters implemented on printed circuit boards (PCB) [1,2,3]. By employing a filter design approach based on cascading high-pass and low-pass sections [4], flexible tuning of the passband range can be achieved. Although such filters can exhibit favorable performance, their overall size remains relatively large due to the limitations of PCB technology, making them difficult to meet the stringent compactness requirements of RF front-end components in highly integrated wireless terminal devices.

Among various miniaturized filter solutions, integrated passive device (IPD) technologies, such as silicon-based IPD processes [5,6] and glass-based IPD processes [7,8], have demonstrated promising application potential in filter design due to their high fabrication accuracy and relatively high quality (Q) factor. In addition, passive integration technologies also include GaAs-substrate-based IPD processes [9,10,11,12], which feature high dielectric constants and low loss, making them particularly suitable for the realization of high-performance RF and microwave passive components. However, filters designed using IPD technologies generally exhibit fixed operating frequency bands, as tuning elements are not integrated on chip, which limits their frequency tunability. To overcome this limitation, several studies have attempted to combine IPD technologies with external tunable components to achieve tunable filter operation. For instance, by integrating IPD filters with Micro-Electro-Mechanical Systems (MEMS) [13], notch filters can be cascaded into fixed-band bandpass filters, enabling the tuning of the notch center frequency within the bandpass filter’s operating range. Additionally, research has explored combining IPD processes with barium strontium titanate (BST) varactors [14]. By replacing the capacitors in the circuit that influence the location of *TZs* with BST varactors, the filter can provide targeted suppression of interference signals at specific frequencies. Beyond on-chip tunable filters based on IPD processes, reconfigurable filters based on the GaAs pseudomorphic high electron mobility transistor (pHEMT) process achieve tuning of the filter’s center frequency or bandwidth. This is accomplished by using the on/off states of pHEMT to adjust the locations where *TZs* are generated [15] or tune shunt-grounded resonators [16] to shift the filter center frequency. Furthermore, CMOS Q-enhanced tunable filters [17,18] incorporate active compensation circuits to effectively mitigate the limited Q factor of passive components, thereby reducing loss and boosting the Q factor to realize highly selective narrowband filters. In addition to active compensation approaches, N-path filters [19,20] can also realize passband tuning and frequency reconfigurability through switch networks operating within the passband.

In this paper, a quasi-lumped element tunable bandpass filter based on a novel GaAs varactor diode process is proposed. In the layout, a quasi-lumped structure is used to replace spiral inductors, and the design is implemented by combining metal-insulator-metal (MIM) capacitors with novel on-chip varactor diodes. A symmetric structure is adopted to simplify the tuning procedure, and source-load coupling is introduced to enhance passband selectivity. The operating frequency of the filter is changed by separately tuning the coupling capacitance and the grounded series LC resonant unit of the filter. The equivalent circuit is provided and analyzed, and the design concept, implementation method, and the corresponding simulation and experimental measurement results are presented. Measurements show that the center frequency can be tuned from 5.4 GHz to 6.2 GHz, achieving continuous tunability of the center frequency.

## 2. Design of the Tunable Filter

### 2.1. Circuit Configuration

**Figure 1:**
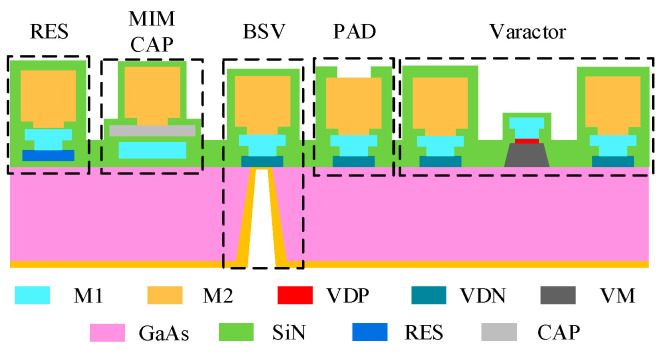
Cross-section of the GaAs IPD process.

The proposed filter in this paper is implemented using GaAs technology. Figure 1 illustrates the stack-up information of the fabrication process used in this design. The process employs a GaAs substrate with a dielectric constant of 12.9 and a thickness of 100 µm. The insulating dielectric layer uses silicon nitride (SiN) with a relative dielectric constant of 6.9. Backside vias (BSV) with a diameter of 50 µm are utilized to provide grounding paths for the circuit, while PADs provide the interface between the circuit and external components. The substrate thickness is 100 µm, and the backside metal thickness is 4 µm.

This process includes two main metal layers, M1 and M2, both made of Gold, with thicknesses of 1 µm and 4 µm, respectively. Therefore, to reduce conductor loss and improve circuit performance, most of the microstrip lines in the circuit are implemented using the thicker M2 layer. The resistor layer (RES) in this process consists of nickel chromium (NiCr) thin-film resistors with a sheet resistance of 50 Ω/square and a thickness of 0.04 µm. The process also provides a capacitor layer (CAP), and the capacitors are implemented using an MIM structure composed of M1/SiN/CAP. The dielectric thickness can be selected as 0.77 µm or 1.85 µm, corresponding to capacitance densities of 720 pF/mm^2^ and 300 pF/mm^2^, with breakdown voltages of 75 V and 150 V, respectively. Considering that the maximum DC voltage in the circuit is 8 V, this work adopts the MIM capacitors with a density of 720 pF/mm^2^ to achieve a larger capacitance within a limited area.

**Figure 2:**
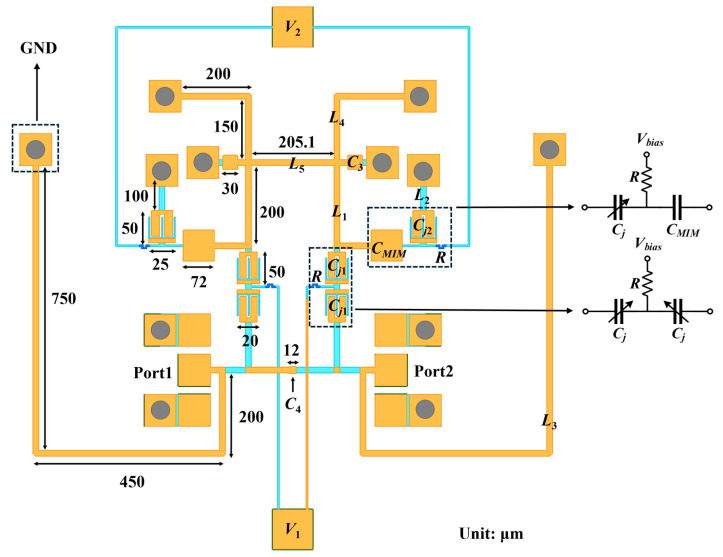
Layout of the proposed filter.

Furthermore, this process integrates PN-junction varactor diodes on-chip. As the bias voltage applied between the n-type metal contact (VDN) and p-type metal contact (VDP) across the varactor mesa (VM) increases, the depletion layer between the PN junctions gradually expands, leading to a decrease in equivalent capacitance. This enables voltage-controlled tuning of the varactor diode’s capacitance [21], providing the foundation for the filter’s tunability. Compared with traditional IPD processes, the on-chip integration of varactor diodes in this process achieves miniaturization of the tunable filter design. Meanwhile, compared to other GaAs-based processes [22], this process employs thicker metal layers, which significantly reduces conductor loss while improving the Q factor of the passive devices. The layout of the proposed quasi-lumped element tunable bandpass filter implemented using this process is shown in Figure 2.

**Figure 3:**
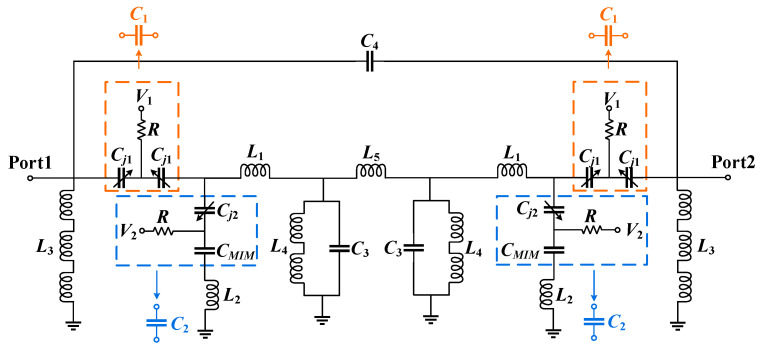
Schematic of the proposed filter.

To facilitate a detailed analysis of the filter operating principle, the structure shown in Figure 2 is equivalently represented by the circuit schematic depicted in Figure 3. The equivalent circuit consists of 9 inductors and 7 capacitors. The two *C_j_*_1_ and resistor *R* are modeled as *C*_1_, while *C_j_*_2_, *C_MIM_*, and resistor *R* are modeled as *C*_2_. Among them, *C*_1_ serves as the coupling capacitor in the circuit. The grounded series LC resonator formed by *C*_2_ and *L*_2_ introduces a TZ in the frequency response. *L*_3_ enhances the stopband rejection on the lower frequency side of the filter, while functioning as a matching circuit in conjunction with the resonant circuit formed by *C*_3_ and *L*_4_. *C*_4_ provides source-load coupling, which improves passband selectivity and enhances out-of-band suppression. In addition, *L*_1_ and *L*_5_ are coupling inductors realized by microstrip-line equivalents in the circuit.

### 2.2. Analysis of the Circuit

According to the circuit shown in Figure 3, the *ABCD* matrix [23] of the schematic without source-load coupling can be expressed as:

(1) $${\left[\begin{array}{cc} A & B\\ C & D \end{array}\right]}_{\mathrm{main}}=\left[\begin{array}{cc} 1 & 0\\ Y_{1} & 1 \end{array}\right]\left[\begin{array}{cc} 1 & Z_{1}\\ 0 & 1 \end{array}\right]\left[\begin{array}{cc} 1 & 0\\ Y_{2} & 1 \end{array}\right]\left[\begin{array}{cc} 1 & Z_{2}\\ 0 & 1 \end{array}\right]\left[\begin{array}{cc} 1 & 0\\ Y_{3} & 1 \end{array}\right]\left[\begin{array}{cc} 1 & Z_{3}\\ 0 & 1 \end{array}\right]\left[\begin{array}{cc} 1 & 0\\ Y_{4} & 1 \end{array}\right]\left[\begin{array}{cc} 1 & Z_{4}\\ 0 & 1 \end{array}\right]\left[\begin{array}{cc} 1 & 0\\ Y_{5} & 1 \end{array}\right]\left[\begin{array}{cc} 1 & Z_{5}\\ 0 & 1 \end{array}\right]\left[\begin{array}{cc} 1 & 0\\ Y_{6} & 1 \end{array}\right]$$

Due to the symmetrical distribution of the schematic, the relationships *Y*_1_ = *Y*_6_, *Z*_1_ = *Z*_5_, *Y*_2_ = *Y*_5_, *Z*_2_ = *Z*_4_, *Y*_3_ = *Y*_4_ are maintained. According to the schematic of the proposed filter, the expressions can be derived as:

(2) $$\begin{array}{c} Y_{1\;}=\;Y_{6\;}=\;\frac{1}{j\omega L_{3}}\\ Z_{1\;}=\;Z_{5\;}=\;\frac{1}{j\omega C_{1}}\\ Y_{2\;}=\;Y_{5\;}=\;\frac{j\omega C_{2}}{1-\omega^{2}C_{2}L_{2}}\\ Z_{2\;}=\;Z_{4\;}=\;j\omega L_{1}\\ Y_{3\;}=\;Y_{4\;}=\;\frac{1-\omega^{2}C_{3}L_{4}}{j\omega C_{3}}\\ Z_{3}=\;j\omega L_{5} \end{array}$$

The coupling capacitor is connected in series between the source and the load of the circuit. The *ABCD* matrix of this source-load coupling path can be expressed as:

(3) $${\left[\begin{array}{cc} A & B\\ C & D \end{array}\right]}_{\mathrm{source}\text{-}\mathrm{load}\;\mathrm{coupling}}=\left[\begin{array}{cc} 1 & \frac{1}{j\omega C_{4}}\\ 0 & 1 \end{array}\right]$$

At this point, the admittance matrix (*Y*-matrix) of the source-load coupling can be expressed as:

(4) $$Y_{\mathrm{source}\text{-}\mathrm{load}\;\mathrm{coupling}\;}=\left[\begin{array}{cc} \frac{D}{B} & \frac{\mathrm{BC}-\mathrm{AD}}{B}\\ -\frac{1}{B} & \frac{A}{B} \end{array}\right]=\left[\begin{array}{cc} j\omega C_{4} & -j\omega C_{4}\\ -j\omega C_{4} & j\omega C_{4} \end{array}\right]$$

Let $Y_{11}^{\prime }$, $Y_{12}^{\prime }$, $Y_{21}^{\prime }$ and $Y_{22}^{\prime }$ denote the admittance matrix parameters of the circuit without source-load coupling; then the total admittance matrix of the circuit can be expressed as:

(5) $${Y=Y}_{\mathrm{main}}+Y_{\mathrm{source}\text{-}\mathrm{load}\;\mathrm{coupling}}=\left[\begin{array}{cc} j\omega C_{4}+Y_{11}^{\prime } & -j\omega C_{4}+Y_{12}^{\prime }\\ -j\omega C_{4}+Y_{21}^{\prime } & j\omega C_{4}+Y_{22}^{\prime } \end{array}\right]$$

The *S*-parameters of the filter can be derived from the circuit’s admittance matrix as:

(6) $$\begin{array}{c} S_{11}=\;\frac{\left(Y_{0}-Y_{11}\right)\left(Y_{0}+Y_{22}\right)+Y_{12}Y_{21}}{\Delta Y}\\ S_{21}=-\frac{2Y_{21}Y_{0}}{\Delta Y} \end{array}$$

In Equation (6), $\Delta Y\;=\;\left(Y_{11}+Y_{0}\right)\left(Y_{22}+Y_{0}\right)-Y_{12}Y_{21}$, $Y_{0}\;=\;1/Z_{0}$, $Z_{0}$ represents the characteristic impedance of the circuit. When *TZs* occur, *S*_21_ = 0. As can be seen from Equation (6), the necessary condition is *Y*_21_ = 0, where *Y*_21_ can be expressed as:

(7) $$Y_{21}\;={\;Y}_{21}^{\prime }-j\omega C_{4}$$

When *Y*_21_ is zero, while the denominator is non-zero and does not approach zero, the condition *S*_21_ = 0 holds, thereby generating *TZs*. When the transfer admittances of the two paths are equal, Equation (8) can be solved to determine the frequency location of the *TZs*.

(8) $$Y_{21\;}^{\prime }=j\omega C_{4}$$

**Figure 4:**
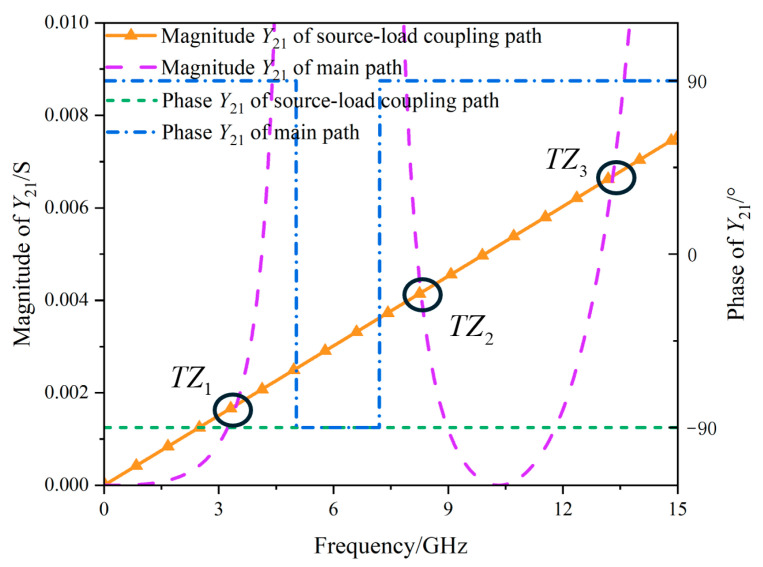
Simulation of transfer admittance curves for the two transmission paths.

Figure 4 illustrates the magnitude and phase of the transfer admittances for the two signal transmission paths. When the magnitudes of the transfer admittances of the two paths intersect and the phase difference between them is 180° at the corresponding frequency, the condition is satisfied. According to Equation (8), *TZs* are formed at this specific frequency.

**Figure 5:**
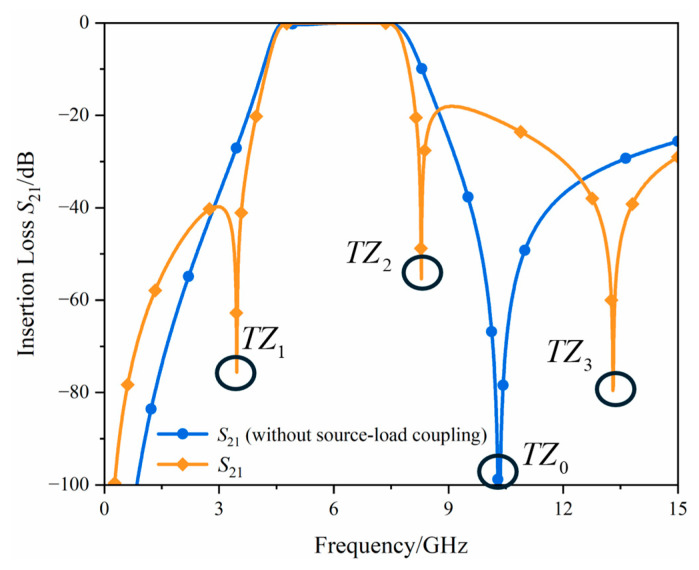
Simulation of the source-load coupling schematic and its corresponding *TZs*.

Figure 5 illustrates the impact of source-load coupling on the circuit. In the absence of source-load coupling, the generated TZ is determined by the grounded resonator, and its corresponding *TZ*_0_ frequency is expressed as:

(9) $$f_{\mathrm{TZ}0}=\frac{1}{2\pi \sqrt{L_{2}C_{2}}}$$

With the addition of the source-load coupling capacitor *C*_4_, the selectivity of the filter passband is enhanced. Meanwhile, the frequency location of *TZ*_0_ shifts toward the passband side, and two additional *TZs* are generated. The component values in the circuit at this point are: *L*_1_ = 0.12 nH, *L*_2_ = 0.15 nH, *L*_3_ = 0.82 nH, *L*_4_ = 0.2 nH, *L*_5_ = 0.005 nH, *C*_1_ = 0.9 pF, *C*_2_ = 1.585 pF, *C*_3_ = 0.4 pF, and *C*_4_ = 0.08 pF.

### 2.3. Tunable BPF Design

**Figure 6:**
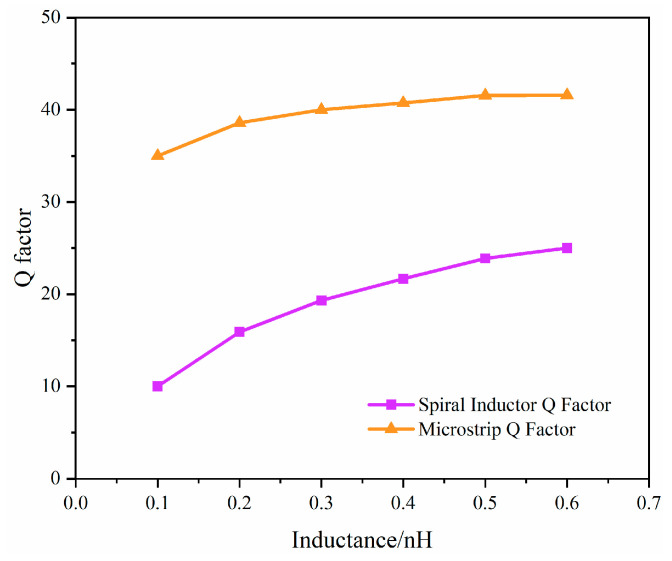
Q factor comparison between microstrip and spiral inductors for identical inductance values at 6 GHz.

Due to technological constraints, the center frequency of filters implemented with lumped elements is often limited to below 6 GHz. Meanwhile, circular inductors demand high fabrication precision and specific dimensions, making it difficult to achieve compact yet high-performance inductor structures. Figure 6 shows a comparison of the Q factors of a microstrip and a circular spiral inductor at 6 GHz, obtained from electromagnetic (EM) simulations under the current process conditions, where both have a line width of 15 µm, across different equivalent inductance values.

As shown in Figure 6, under the condition of the same equivalent inductance value, the Q factor of the microstrip-implemented inductor is significantly higher than that of the spiral inductor. Furthermore, microstrip can achieve relatively smaller inductance values, providing greater design flexibility. Based on these advantages, microstrip is employed in the layout design to realize the inductors in the topology.

**Figure 7:**
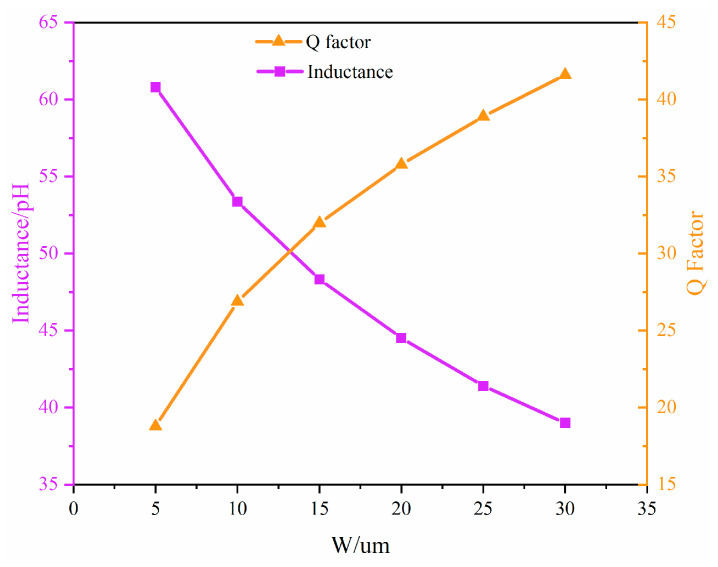
EM simulation of equivalent inductance and Q factor under different microstrip widths at 6 GHz.

The Q factor of microstrip is affected by the skin effect at high frequencies [23]. As the frequency increases, the current becomes more concentrated on the metal surface. In this case, increasing the conductor cross-section can significantly increase the effective conducting area, thereby reducing conductor loss to enhance the Q factor. Figure 7 shows the EM simulation results of the equivalent inductance and its Q factor versus line width at 6 GHz for a 100-µm-long microstrip line. As can be seen from the figure, the inductance value decreases as the width increases, while the Q factor increases with width. A smaller inductance per unit length implies that a larger layout area is required to achieve the same equivalent inductance, whereas a higher Q factor corresponds to lower loss. To balance size and performance, the inductors in the topology are implemented using microstrip lines with a width of 15 µm.

Once the microstrip width *W* and the substrate thickness *h_Sub_* are determined, the characteristic impedance *Z*_01_ of the microstrip can be obtained as [23]:

(10) $$\begin{array}{c} Z_{01}=\frac{60}{\sqrt{\varepsilon_{e}}}ln\left(\frac{8h_{\mathrm{Sub}}}{W}+\frac{W}{4h_{\mathrm{Sub}}}\right)\\ \varepsilon_{e}=\frac{\varepsilon_{r}+1}{2}+\frac{\varepsilon_{r}-1}{2}\frac{1}{\sqrt{1+12h_{\mathrm{Sub}}/W}} \end{array}$$

In Equation (10), $\varepsilon_{r}$ represents the relative permittivity, and θ is the electrical length. The input impedance of the short-circuited microstrip can then be expressed as:

(11) $$Z_{\mathrm{in}}=jZ_{01}\tan\theta$$

At this point, the equivalent inductance *L_eq_* can be calculated as:

(12) Leq=Im(Zin)ω

Additionally, at 6 GHz, the guided wavelength $\lambda_{g}$ is calculated to be approximately 13.9 mm, whereas the longest microstrip-based equivalent inductor in Figure 2 is 1.4 mm. This length is significantly smaller than $\lambda_{g}/4$, satisfying the quasi-lumped-element design criterion. At this point, the microstrip dimensions can be determined based on the inductance values in the topology, providing a reference for the layout design. The inductor *L*_2_ in the layout is connected to the varactor diode and it is implemented using the M1 layer, all other inductors are implemented using the M2 layer.

**Figure 8:**
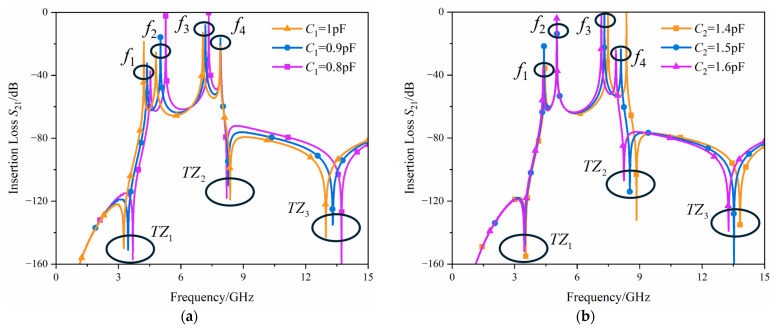
Resonance frequency versus *C*_1_/*C*_2_ variation curves (**a**) *C*_1_; (**b**) *C*_2_.

Figure 8 illustrates the impact of *C*_1_ and *C*_2_ on the filter frequency response under weak coupling conditions. In the schematic, *C*_1_ serves as the coupling capacitor in the circuit. By adjusting *C*_1_, the coupling of the filter can be controlled. As shown in Figure 8a, setting *C*_1_ as a tuning element allows the filter bandwidth to be adjusted.

Meanwhile, *C*_2_ is a constituent part of the TZ structure in the filter. According to Equation (9), the location of the TZ can be modified by adjusting *C*_2_. The frequency of the TZ location is inversely proportional to the capacitance value within the TZ structure. Similarly, as shown in Figure 8b, a smaller *C*_2_ value causes the filter’s resonance frequency to shift toward the upper sideband. When the coupling capacitor *C*_1_ is tuned, the filter bandwidth changes and both the lower and upper passband edges shift accordingly. By then tuning *C*_2_ to adjust the upper passband edge, the operating bandwidth of the filter can be maintained at a constant value. In this process, the resonance frequencies interact with each other, thereby achieving tuning of the filter’s operating frequency, by tuning these two capacitors, the filter can achieve continuous adjustability within its operating frequency range. Thus, *C*_1_ and *C*_2_ are determined as the tuning elements, enabling the adjustability of the filter’s operating frequency by changing its resonance frequency.

**Figure 9:**
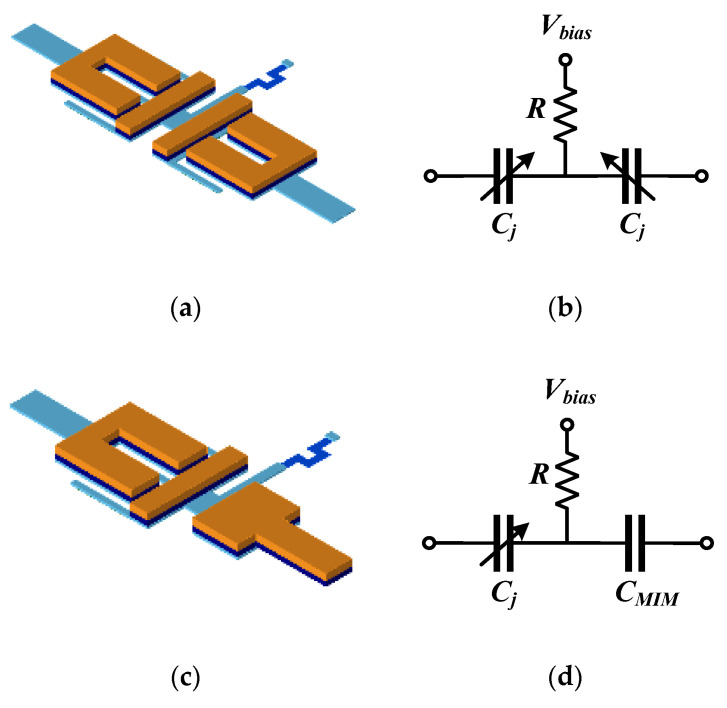
Varactor diode structures (**a**) Layout of Type A; (**b**) Schematic of Type A; (**c**) Layout of Type B; (**d**) Schematic of Type B.

Introducing varactor diodes into a passive circuit brings inherent nonlinearity. Meanwhile, limited by the reverse-bias voltage and affected by self-heating due to the parasitic series resistance, the devices are prone to temperature drift and may face risks such as thermal breakdown, which significantly reduces the power-handling capability of the circuit. Based on these considerations and the tuning requirements for different capacitance values, this work designs two different varactor tuning structures. Both ends of this structure serve as input/output ports. A current-limiting and DC-blocking resistor is connected to the biasing point, with the bias voltage applied to the other side of the resistor. To achieve an optimal frequency tuning range, the two different varactor diode structures are analyzed. Figure 9a shows Type A, which is a series structure of two 20 µm × 30 µm varactor diodes, and Figure 9b is its equivalent circuit diagram. Figure 9c shows Type B, which consists of a 20 µm × 30 µm varactor diode in series with an MIM capacitor, and Figure 9d is its equivalent circuit diagram.

**Figure 10:**
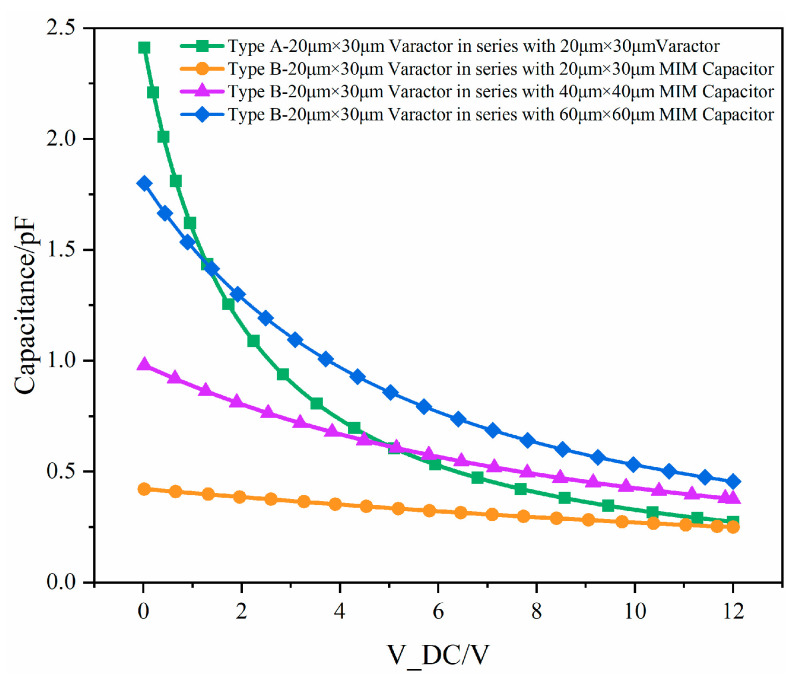
C-V characteristic curve simulations for two different varactor diode structures.

The equivalent capacitance of the varactor diode can be expressed [24] by Equation (13), where *C_j_*_0_ is the junction capacitance of the varactor diode without bias voltage, *V* is the bias voltage, *V_D_* is the built-in potential, and *n* is the nonlinearity coefficient of the varactor diode, which is related to the PN junction structure and doping profile. As indicated by the formula, the junction capacitance exhibits a nonlinear decreasing trend with the reverse bias voltage. Figure 10 shows the C-V curves of the two different varactor diode structures at a frequency of 6 GHz. According to the simulation, compared with Type B, Type A possesses a wider capacitance tuning range. Conversely, the smaller capacitance variation range of Type B under the same voltage conditions can reduce measurement errors caused by direct current (DC) jitter.

Based on Figure 8 and the component values in the schematic, since *C*_1_ requires a larger capacitance tuning range, *C*_1_ adopts a Type A configuration, with two 20 μm × 50 μm varactor diodes connected in series. *C*_2_ adopts a Type B configuration, with a 25 μm × 50 μm varactor diode connected in series with a 72 μm × 72 μm varactor diode. The resistors in both branches are set to 4 μm × 45 μm, corresponding to an equivalent resistance of 562.5 Ω.

(13) $$C_{j}=\frac{C_{j0}}{{\left(1+\frac{V}{V_{D}}\right)}^{n}}$$

## 3. Measurement and Discussion

**Figure 11:**
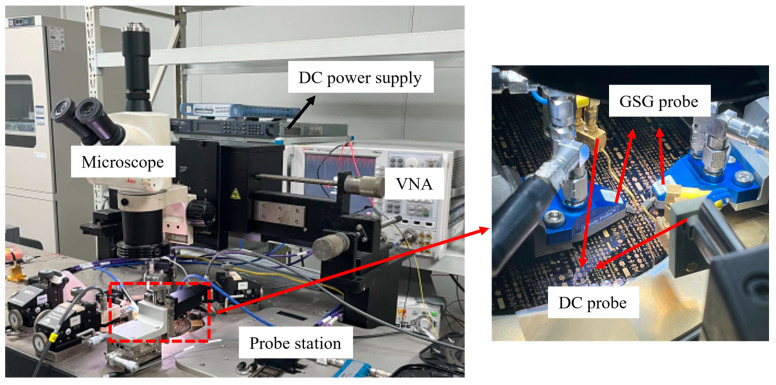
Measurement setup.

Figure 11 shows the measurement setup used in this work. The measurements were carried out on a probe station. The bias voltages were supplied by a DC power supply with two independent output channels for tuning V1 and V2. Two DC probes were connected to the power supply to apply two independent bias voltages to the filter. The S-parameters were measured using a vector network analyzer (VNA) with two 100-µm-pitch ground-signal-ground (GSG) probes.

**Figure 12:**
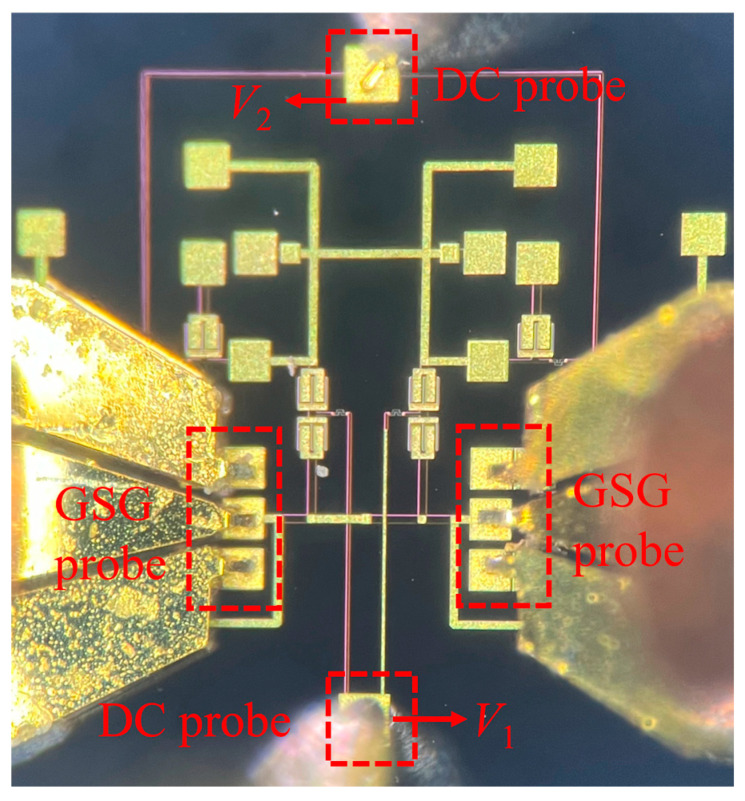
Photograph of the proposed fabricated filter.

To verify the design of the proposed tunable filter, Figure 12 shows the fabricated filter. The total chip area is 1.35 × 1.365 mm^2^. To simplify the measurement procedure, the symmetric varactor diodes are connected to the same pad using thin microstrip lines. Two bias pads are placed on the top and bottom sides of the chip to facilitate voltage application using DC probes.

**Figure 13:**
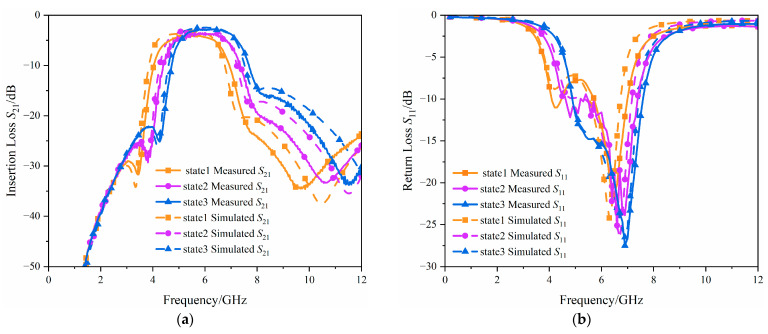
Simulated and measured results of the filter (state 1: *V*_1_ = 4 V, *V*_2_ = 4.5 V, state 2: *V*_1_ = 5 V, *V*_2_ = 5.6 V, state 3: *V*_1_ = 6.8 V, *V*_2_ = 7 V) (**a**) *S*_21_; (**b**) *S*_11_.

A comparison between simulation and measurement results is shown in Figure 13. The simulations included the interconnects and pads, and electromagnetic parameters were extracted using Momentum in Advanced Design System (ADS), followed by EM co-simulation (emCosim) for combined simulation. The equivalent capacitance of the GaAs varactor diode is susceptible to temperature drift. To ensure consistency between the measurements and the simulation conditions, and to minimize the impact of temperature variations on the varactor characteristics, both the simulation and measurement were performed at an ambient temperature of 25 °C.

The measurement results indicate that the filter generates three *TZs* within 0–12 GHz. When the circuit operates in three different states, the center frequency can be tuned from 5.4 GHz to 6.2 GHz, with insertion loss varying between 2.5 dB and 3.7 dB, while the absolute bandwidth remains at 2.5 GHz. This indicates that the selected resistor value is reasonable in the varactor structure design. The losses mainly originate from substrate loss, mutual coupling between adjacent components, and the internal equivalent circuit of the components. As the bias voltage increases, the Q factor of the varactor diode increases, therefore, within the operating band, the insertion loss decreases with increasing voltage.

There is some discrepancy between the simulated and measured results, but the overall deviation is small. Specifically, the measured insertion loss is slightly higher than that in simulation, while the measured return loss is better than the simulated result. This is because, although calibration is performed during the measurements to make the measured data as consistent as possible with the actual reference plane of the device, non-ideal effects such as probe-contact parasitics are still unavoidable compared with the simulation model. With the short-open-load-through (SOLT) calibration adopted in this paper, the calibration plane is typically shifted to the probe tips, so the measured results still include the parasitic loss introduced by the probe contact as well as the loss from the probe tips to the device under test, whereas these losses are often not considered in simulations. These factors are the primary reasons for the discrepancies between the simulated and measured results. Table 1 presents a comparison between previously reported tunable filters based on integrated process technologies and the tunable filter proposed in this work. In comparison, the proposed filter features a compact size, a simple tuning scheme, and continuous tuning over the operating frequency band.

## 4. Conclusions

This paper proposes a tunable bandpass filter based on GaAs technology. The filter employs a novel on-chip varactor diode structure, and source-load coupling is introduced in the topology to enhance passband selectivity. In the layout implementation, microstrip structures are employed to replace low-value inductors, thereby improving the filter performance while enabling small inductance values that cannot be readily realized using lumped elements. Measurement results show good agreement with simulations. The filter achieves continuous tuning of the center frequency within a compact chip area, providing an effective design approach for miniaturized tunable filters.

## Figures and Tables

**Table 1:** Performance comparison with prior works.

Reference	[13]	[14]	[15]	[16]	This Work
Process	Silicon IPD	HR-Si IPD ^1^	GaAs pHEMT	GaAs pHEMT	GaAs Varactor
Tuning element	MEMS	BST Varactor	pHEMT	pHEMT	Varactor
Tuning state	Continuous	Continuous	Discrete	Discrete	Continuous
Tuning parameter	Notch frequency	TZ	BW ^2^	Fc ^3^	Fc ^3^
*TZs*	NA ^4^	3	2	0	3
Tuning range (GHz/%)	5.25–5.8	2.3–2.6	14.3–23.5	8.8–11.32	5.4–6.2
IL ^5^ (dB)	2.55–3.86	4–5	2.2	4.1	2.5–3.7
Size ($\lambda_{0}^{2}$)	0.096 × 0.058	0.031 × 0.0125	0.028 × 0.032	0.027 × 0.036	0.027 × 0.0273

^1^ High-Resistivity Silicon; ^2^ Bandwidth; ^3^ Frequency center; ^4^ Not available; ^5^ Insertion loss.

## Data Availability

The original contributions presented in this study are included in the article. Further inquiries can be directed to the corresponding author.
